# Supplementary material for: A Probabilistic Approach to Explore Signal Execution Mechanisms With Limited Experimental Data
Source: Front Genet. 2020 Jul 10;11:686. doi: 10.3389/fgene.2020.00686 (PMC7381302; doi:10.3389/fgene.2020.00686)
Supplement: Supplementary file 7 [file Presentation_1.PDF]

# Supporting Information for

## A probabilistic approach to explore signal execution mechanisms

Michael A. Kochen and Carlos F. Lopez

Carlos F. Lopez.
E-mail: c.lopez@vanderbilt.edu

This file includes: Figures S1 to S4, and a tutorial section.

Figure S1: Model calibration to existing FRET data for cleaved Bid, exported Smac, and cleaved PARP.
Figure S2: Log expected values for the six decomposed extrinsic apoptosis networks for increasing values of XIAP.
Figure S3: Apoptotic signal flux over ranges of XIAP and Bcl-2.
Text S1: Interpretation of the data in Figure S3.
Figure S4: Expected value plots at increasing levels of live points (nested sampling population) for the direct caspase pathway, mitochondrial pathway and complete network.

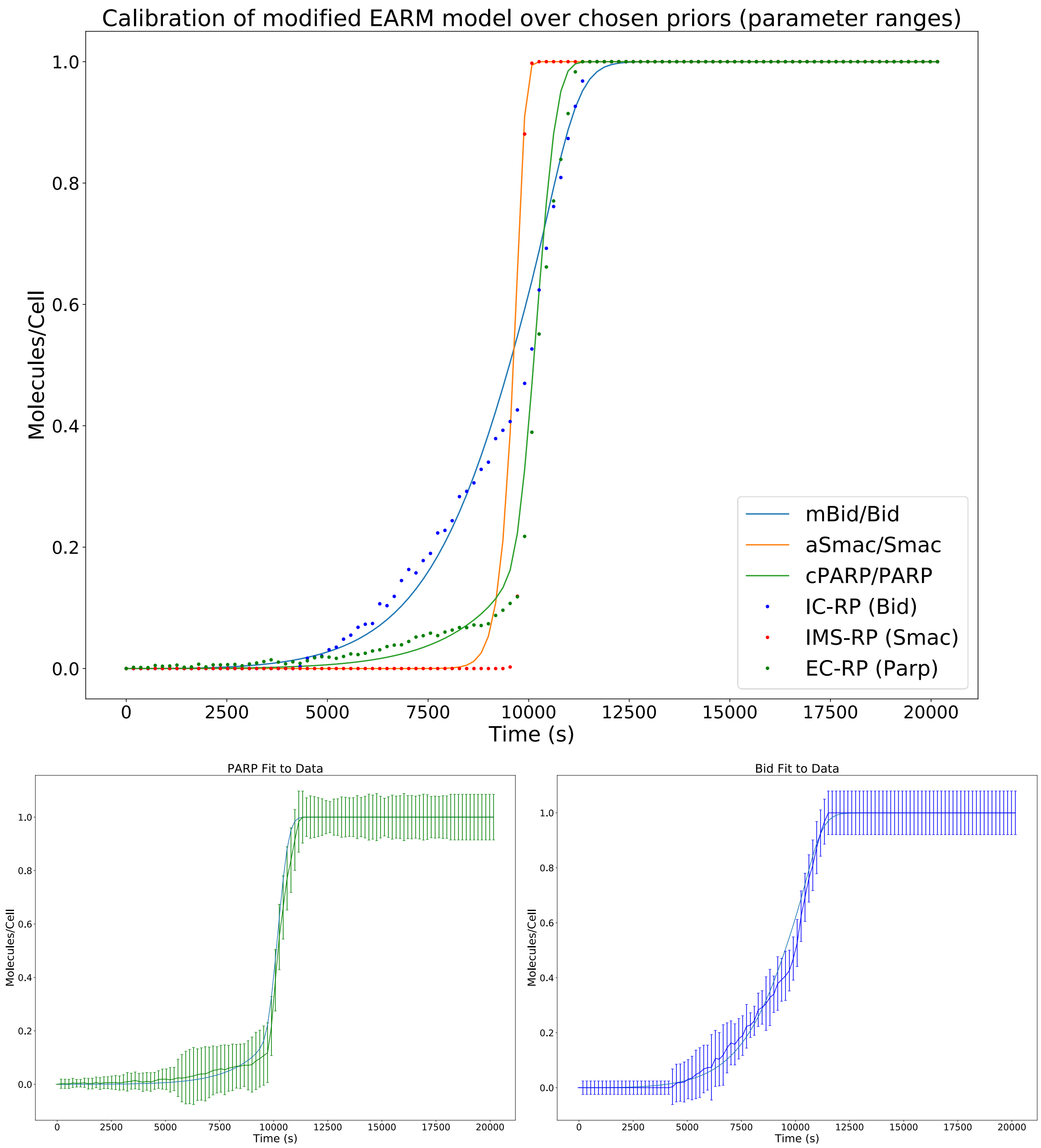


**Figure S1.** (A) Trajectories of cleaved Bid, exported Smac, and cleaved PARP (solid lines) for the calibrated EARM model along with the data used for the calibration (points). (B)-(C) Parp and Bid trajectories respectively along with estimated errors (bars) for the FRET data they were calibrated to.

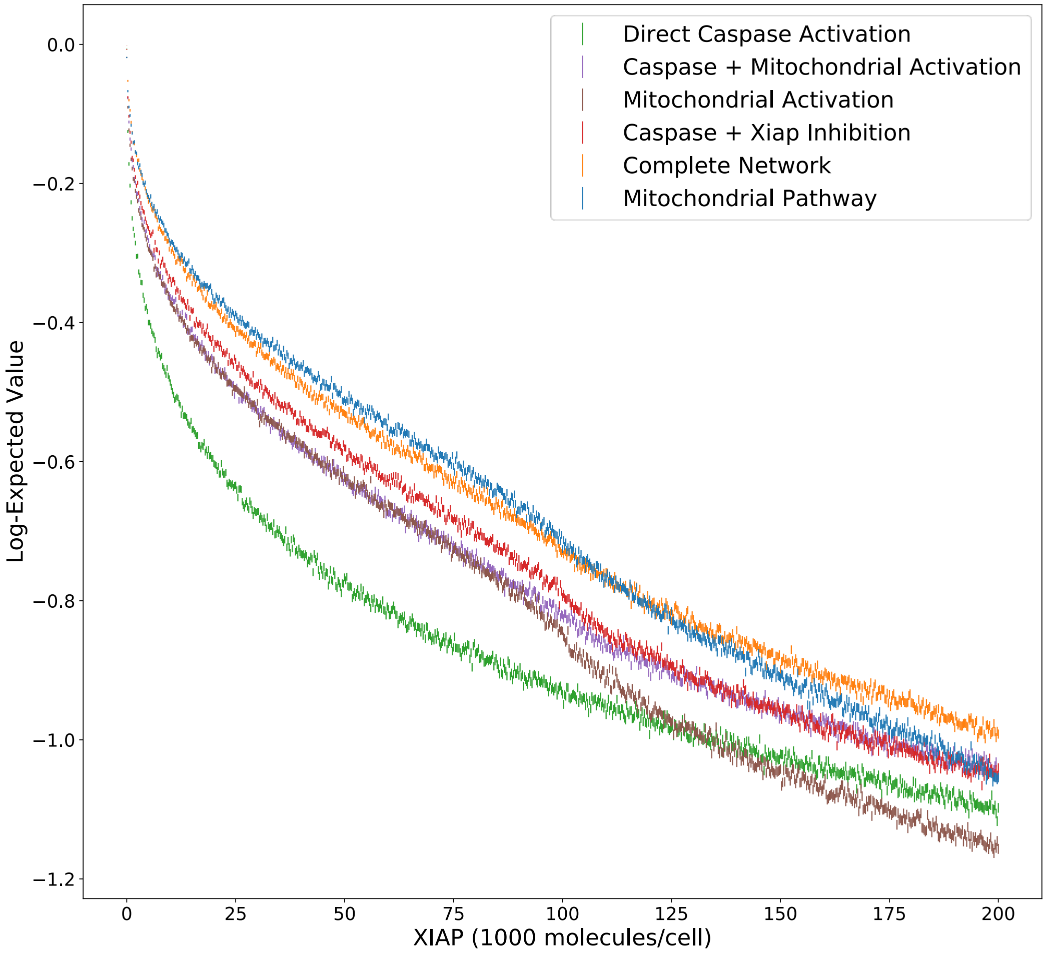


**Figure S2.** log-expected value version of plots in Figure 2G with estimated errors generated by MultiNest.

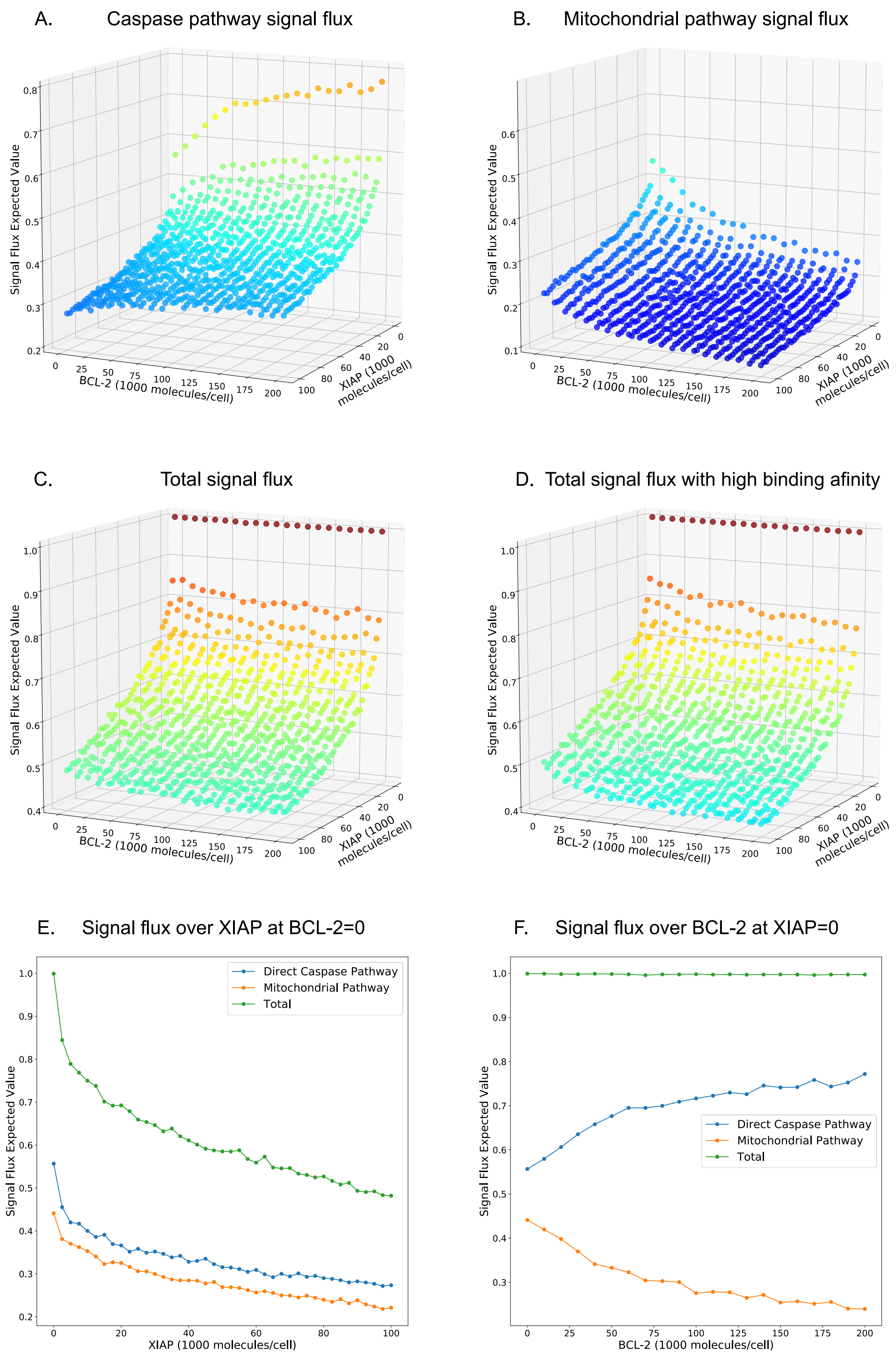


**Figure S3. Apoptotic signal flux over ranges of XIAP and Bcl-2.** (A)-(C) Apoptotic signal flux expected value plots over the apoptosis regulators XIAP (range of 0 to 100,000 molecules per cell) and Bcl-2 (range of 0 to 200,000) for (A) the caspase pathway, (B) the mitochondrial pathway, (C) the total signal flux, and (D) the total signal flux with binding affinities for Bcl-2 and Bad in physiological ranges. (E) and (F) Comparison of the expected value trends at Bcl-2 and XIAP levels of 0 (E and F respectively).

**Text S1. Interpretation of the results from Figure S4.**

Deconstruction of a network into all relevant subnetworks and comparison of the relative changes in apoptosis signal transduction as regulatory conditions change provides a reductionist view of how various network components interact with one another and affect the overall signaling dynamics. To get a holistic view of changes in signaling dynamics that incorporates every network component we calculate the expected values for signal flux through both the caspase and mitochondrial pathways while retaining the complete network model. Instead of considering different models with the same objective function this method considers the same network but equivalent objective functions for different pathway target. Inference of differential signal flow via calculation of pathway flux was introduced in Shockley et al. [1]. In that work they calculated the path fluxes for a number of initial conditions and over an ensemble of parameter sets. Nested sampling, along with a signal flux-based objective function, extends this idea with the calculation of the expected value for signal flux through a target pathway via integration over a prior parameter range. Because both pathways cleave Caspase-3, which goes on to cleave the final product PARP, the objective for each pathway was the sum, over time *T* in seconds, of the proportion of Caspase-3 cleaved through that pathway at time *t* multiplied by the amount of PARP cleaved from time *t-1* to time *t* (see Methods). In addition to runs for both pathways an additional run for the total signal flux was carried out. We explored an XIAP concentration ranging from 0 to 100,000 molecules per cell and Bcl-2 from 0 to 200,000 molecules per cell, in increments of 2500 and 10,000 respectively, producing a 3-dimensional expected value landscape for each target objective. Because the computational cost when using these objective functions is significantly higher than simply calculating the proportion of cleaved PARP at the end of a simulation, the number of live points in the sampling algorithm was reduced from 16,000 to 4,000.

The expected value for signal flux through the caspase pathway showed a sharp initial decline that becomes more gradual as XIAP increases (Figure S4A). This appears to be more pronounced at the higher end of the Bcl-2 range. As Bcl-2 is increased the expected values increase, again becoming more gradual at higher levels of Bcl-2. This effect is clearly more prominent for lower levels of XIAP, likely because of the overall higher levels of signal throughput. The increase in signal flux through the caspase pathway as Bcl-2 increases is indicative of a shifting apoptotic signal from the mitochondrial to the caspase pathway; this is also evident in the decreasing mitochondrial signal flux as Bcl-2 increases (Figure S4B). As with the caspase pathway, increasing XIAP levels causes a decrease in signal flux through the mitochondrial pathway. The effects of both Bcl-2 and XIAP appear to diminish as the other increases. The combined responses to increases in XIAP and Bcl-2 for the caspase and mitochondrial pathways are evident in the expected value landscape for total flux. The total apoptotic signal flux decreases sharply as XIAP increases but remains largely stable, with only relatively small declines, as Bcl-2 is increased. Thus, while XIAP inhibits the flux through both pathways, Bcl-2, under the given simulation conditions, appears to primarily shift the flux to the caspase pathway and inhibits apoptosis to only a small degree compared to XIAP. Note that the expected values for signal flux through the caspase and mitochondrial pathways are additive. The average and average absolute differences between the total flux and the combined caspase and mitochondrial flux are -0.00565 and 0.00866 respectively (Table S4).

Figure S4E displays the expected value trends for each objective over the full range of XIAP and at a Bcl-2 level of 0 for a fully active mitochondrial pathway. Throughout the range of XIAP (and Bcl-2 as well, Figures S4A and S4B), the caspase pathway retains a consistently higher signal flux. This supports the hypothesis that a significant proportion of the mitochondrial signal amplification is due to facilitation of the signal through the caspase pathway via XIAP inhibition and may in fact be the primary mechanism. Figure S4F displays the expected value trends over the range of Bcl-2 at an XIAP level of 0, which clearly

shows a shift in flux from the mitochondrial to the caspase pathway. Total flux is nearly complete throughout the range, meaning that the signal results in the cleavage of nearly all available PARP. The shift in signal flux from the mitochondrial to the caspase pathway appears to be, at every level of XIAP and under these simulation conditions, the primary effect of Bcl-2. This implies that even a weak signal through the mitochondria may be enough to inhibit XIAP via SMAC and that much higher binding affinities than the given parameter ranges allowed would be required for Bcl-2 to have a substantial inhibitory effect on apoptosis. Shifting the ranges for the rates of Bcl-2:Bid and Bcl-2:Bax dissociation to [-8.0, -4.0] and [-7.0, -3.0] respectively brings the $K_d$ values roughly in line with [2] and does indeed produce a more pronounced, but still modest, effect (Figure S4D). Further adjustments of the model, both in the initial values and parameter ranges, may be necessary to elicit a higher impact from Bcl-2.


1. Shockley EM, Rouzer CA, Marnett LJ, Deeds EJ, Lopez CF. Signal integration and information transfer in an allosterically regulated network. npj Syst Biol Appl. 2019 Jul 18;5(1):1–9.

2. Ku B, Liang C, Jung JU, Oh B-H. Evidence that inhibition of BAX activation by BCL-2 involves its tight and preferential interaction with the BH3 domain of BAX. Cell Res. 2011 Apr;21(4):627–41.

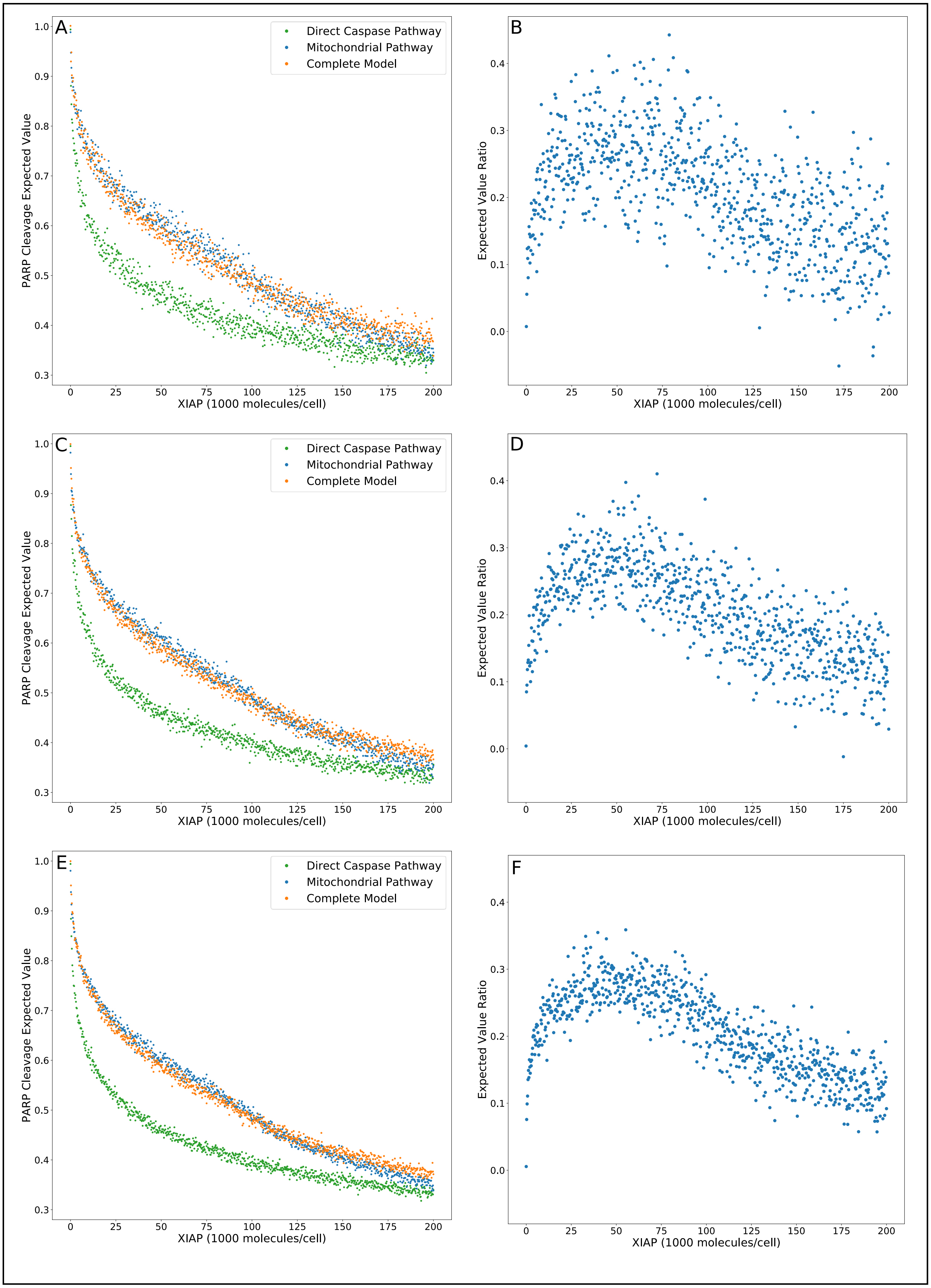
A
B
C
D
E
F
Direct Caspase Pathway
Mitochondrial Pathway
Complete Model
PARP Cleavage Expected Value
Expected Value Ratio
XIAP (1000 molecules/cell)

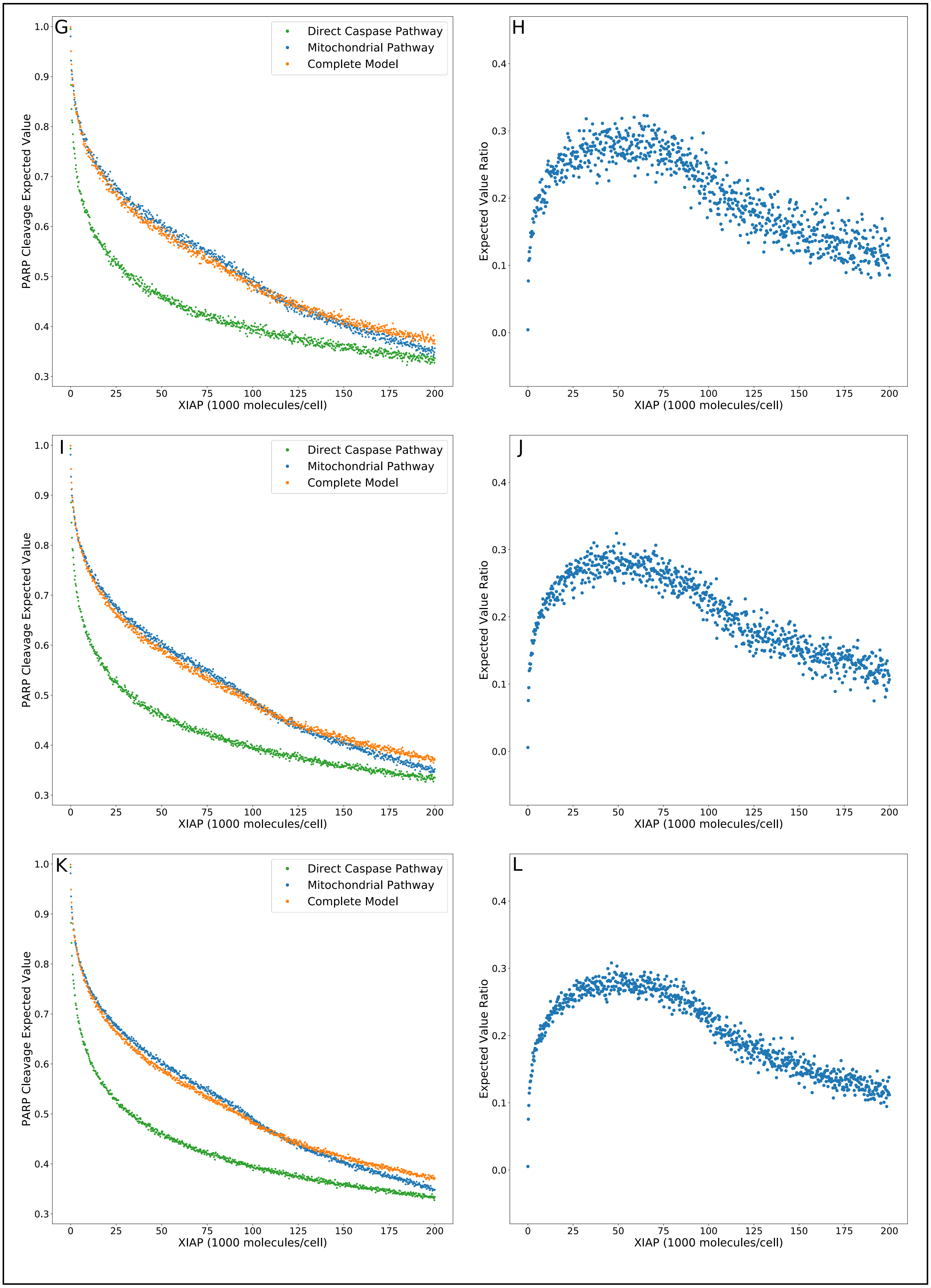

G
Direct Caspase Pathway
Mitochondrial Pathway
Complete Model
PARP Cleavage Expected Value
XIAP (1000 molecules/cell)
H
Expected Value Ratio
XIAP (1000 molecules/cell)
I
Direct Caspase Pathway
Mitochondrial Pathway
Complete Model
PARP Cleavage Expected Value
XIAP (1000 molecules/cell)
J
Expected Value Ratio
XIAP (1000 molecules/cell)
K
Direct Caspase Pathway
Mitochondrial Pathway
Complete Model
PARP Cleavage Expected Value
XIAP (1000 molecules/cell)
L
Expected Value Ratio
XIAP (1000 molecules/cell)

**Figure S4.** (A), (C), (E), (G), (I), and (K), expected value plots over increasing levels of XIAP for the direct caspase (green), mitochondrial (blue), and complete (orange) networks with nested sampling population levels of 500, 1000, 2000, 4000, 8000, and 16,000 respectively. (B), (D), (F), (H), (J), and (L), complete/caspase expected value ratio plots derived from the respective expected value plots in (A), (C), (E), (G), (I), and (K).

## Probabilistic Modeling Tutorial

How to use the Bayesian evidence estimation software MultiNest with the rule-based biological modeling software PySB to produce expected values for quantities of interest from simulations of biological systems.

1. Concept and Overview

The goal of probabilistic modeling is to estimate expected values for biological quantities of interest from physicochemical models of biological systems when a lack of data renders explicit parameter calibration too uncertain to be relied upon. From a modeling perspective we represent quantities of interest as objective functions on the simulated model output. Because the parameters are too uncertain to rely on any one set of values, we average the simulated quantitative outcomes over the multi-dimensional parameter distribution that best represents the data we do have and/or any assumptions we may make about those parameters. This produces expected values for those quantities that can be compared upon in silico perturbations just as explicit quantitative values can be when the parameter values are known.

2. Software

The software required to compute expected values for quantities of interest from biological models consist of a method for integrating our objective function over a given parameter distribution and physicochemical simulator for computing that function. Many potential setups exist. Here we use MultiNest as our function integrator, PySB as our simulator, and PyMultiNest, Python wrapper for MultiNest, to for easy integration of the two. Please visit their respective repositories for downloads and installation instructions.

2.1 MultiNest

MultiNest s designed for the calculation of Bayesian evidence for the purpose of model selection from a set of competing hypothetical models. It used nested sampling along with ellipsoidal clustering to efficiently make this calculation. MultiNest software and installation instructions can be found at https://github.com/JohannesBuchner/MultiNest.git.

2.2 PySB

PySB is a Python-based framework for rule-based modeling and simulation of physicochemical models. PySB software and installation instructions can be found at http://pysb.org.

2.3 PyMultiNest

PyMultiNest is a Python wrapper for MultiNest and enables easy integration of MultiNest and PySB. PyMultiNest software and installation instructions can be found at https://johannesbuchner.github.io/PyMultiNest. A forked version of PyMultiNest that keeps track of failed simulations can be found at https://github.com/LoLab-VU/PyMultiNest.

## 3. Run Files

The run files integrate model simulation by PySB into the sampling of MultiNest via the PyMultiNest wrapper. This can be broken down into four parts: (1) importing a model and creating a PySB solver object for it, (2) defining the prior parameter distributions, (3) defining the simulation based objective function, and (4) running the MultiNest sampler via the PyMultiNest interface.

### 3.1 Model solver object instantiation

Here we assume that a model has previously been constructed in the PySB format. PySB can also import models from various formats. For instructions on how to create a model see the PySB documentation at https://pysb.readthedocs.io/en/stable/index.html. Suppose we have a model defined in model_0.py. We can set up the model Solver with the following lines in the run file run_model_0.py:

```
from model_0 import model
time = [t1, t2, … tn]
model_solver = Solver(model, time)
```

Here, `time` sets the time in seconds for which the simulation will run and provides a list of time points that will be available for inspection after the completion of model simulation. Simulation is initiated with `model_solver.run(point)` where `point` is a provided parameter set, i.e., a point within parameter space. This sets up `model_solver` to be simulated repeatedly with model parameter values sampled from the prior distribution and within the objective function passed to MultiNest (see below). Several additional integrator/simulation methods are available in PySB and we would direct you to the PySB documentation for further exploration.

### 3.2. Defining Priors

The priors are normalized parameter distributions from which MultiNest will sample. The prior is defined as a transformation from the unit cube to the desired parameter space. In this work that definition takes the form:

```
def prior(cube, ndim, nparams):

    for k, every in enumerate(model.parameters):
        if every.name[-3:] == '1kf':
            cube[k] = cube[k]*4 - 4
        if every.name[-3:] == '2kf':
            cube[k] = cube[k]*4 - 8
        if every.name[-3:] == '1kr':
            cube[k] = cube[k]*4 - 4
        if every.name[-3:] == '1kc':
            cube[k] = cube[k]*4 - 1
```

There are four types of reactions in the model and the parameters for them are distinguished by their postfix as follows: 1st order forward: 1kf, 2nd order forward: 2kf, 1st order reverse: 1kr, and catalytic: 1kc. We defined the distribution for each of the types of reactions as a uniform distribution four orders of magnitude in width and centered around values deemed plausible. Sampling from $\log_{10}$ space we the shift and stretch the unit cubes accordingly. For example, every 2st order forward reaction (2kf) will have a parameter value between $10^{-8}$ and $10^{-4}$. As we want to sample from log space, we expand the unit cube [0, 1] by 4 and shift it by -8 to obtain [-8, -4] for this type of reaction. The other reactions are handled similarly. If data for parameter calibration is available transformation to more informative distributions can be made. Note that `ndims` and `nparams` are not used in this prior or in the objective function below. They are passed to PyMultiNest and substituted in when pymultinest.run() is called. We will define the number of dimensions below and PyMultiNest will internally set the two parameters as equal.

3.4. Defining Objective Functions

To iterate over and sample the reaction rate parameters, we use the `model.parameters` construct in PySB. However, initial values are also in this list, so we must first be able to identify the reaction rate parameters from that list. This is easily done using a list of postfixes as defined in the prior. The postfixes are thus defined as

```
postfixes = ['1kf', '2kf', '1kr', '1kc']
```

Although we are replacing likelihood functions with objective functions, we choose to conform to the formatting used in the PyMultiNest documentation. Hence, we define our objective functions as `loglike` functions. The objective function for the quantity of PARP cleaved at the end of a simulation and for a model of the direct caspase pathway is given below and incorporates the following basic steps.

1. Iterate through the reaction rate parameters, sample x from the appropriate uniform distribution, and add $10^x$ to the current list parameters that will represent a sample point from parameter space.
2. Run the model Solver on that point from parameter space. For example, `model_solver.run(point)`
3. Check that the values of the simulation output are within the proper constraints and are numerical values. This is done because wide parameter sampling of complex models can easily lead to failed simulations.
4. If the simulation succeeds return the ratio of cleaved PARP to Total PARP. If it fails return a value that is set below a threshold that MultiNest will ignore and move on to the next sample. The number of successful and failed simulations is also tracked. The forked version of PyMultiNest is assumed here.

```
def loglike(cube, ndim, nparams):
      # 1
      point = []               # point in parameter space
      cube_index = 0           # parameter distribution index

      # Iterate over model parameters and build the parameter list by
      # sampling from the appropriate prior distribution.

      for k, every in enumerate(model.parameters):
            if every.name[-3:] in postfixes:
                  point.append(10**cube[cube_index])
                  cube_index += 1
            else:
                  point.append(model.parameters[k].value)

      # 2
      # Simulate the model with the constructed parameter list.
      model_solver.run(point)

      # 3
      # Verify if simulation was successful.
      failed = False
      for every in model_solver.yobs:
            for thing in every:
                  if thing <= -0.00000001 or np.isnan(thing):
                        failed = True

      # 4
      # Calculate and return the proportion of cleaved PARP to total PARP if
      # successful. Return below threshold value if failed. Also track failed
      # and successful simulations.

      if failed:
            return ['fail', -10000.0]
      else:
            # model_solver.yobs provides simulated values for observables in
            # the model, such as cleaved [12] and uncleaved [2] PARP.

            parpc = model_solver.yobs[-1][12]/(model_solver.yobs[-1][2] +
            model_solver.yobs[-1][12])
            if (parpc > 0.0) and (parpc < 1.00000001):
                  print log(parpc), point
                  return ['sim', log(parpc)]
            else:
                  return ['fail', -10000.0]
```

Note that objective functions (and run files in general) are specific to a model, the target quantity, and the target timepoint. Each will need to be individually tailored.

3.5. Run PyMultiNest

To run PyMultiNest we need to have a count of the parameters. We can do that with the following:

```
n_params = 0
for m, lotsa in enumerate(model.parameters):
      if lotsa.name[-3:] == '1kf':
            n_params += 1
      if lotsa.name[-3:] == '2kf':
            n_params += 1
      if lotsa.name[-3:] == '1kr':
            n_params += 1
      if lotsa.name[-3:] == '1kc':
            n_params += 1
```

The objective function and forked version of PyMultiNest are constructed to count the number of successful and failed simulation. Thus, we set up a counter with.

```
counts = [0, 0]
```

We then run PyMultiNest with

```
pymultinest.run(loglike, prior, n_params,
outputfiles_basename='/path/to/output', counts=counts)
```

There are several other parameters for `pymultinest.run` and we would suggest consulting that software's documentation. For each model series directory in the repository https://github.com/LoLab-VU/BIND is a write.py utility that will generate run files for every model in that directory.
